# Retinal transduction profiling of diverse AAV serotypes via intravitreal injection

**DOI:** 10.1128/jvi.00637-25

**Published:** 2025-08-12

**Authors:** Tianlu Zhang, Fei Wang, Yang Wu, Jingjing Cao, Yin Shen

**Affiliations:** 1 Eye Center, Renmin Hospital of Wuhan University 117921, Wuhan, Hubei, China; 2 College of Life Science and Chemistry, Hunan University of Technology 12572, Zhuzhou, Hunan, China; 3 State Key Laboratory of Magnetic Resonance Spectroscopy and Imaging, Wuhan Center for Magnetic Resonance, Innovation Academy for Precision Measurement Science and Technology, Chinese Academy of Sciences 566813 , Wuhan, Hubei, China; 4 Zhongmou Therapeutics, Wuhan, Hubei, China; 5 Frontier Science Center for Immunology and Metabolism, Medical Research Institute, Wuhan University 619779, Wuhan, Hubei, China; Cornell University Baker Institute for Animal Health, Ithaca, New York, USA

**Keywords:** adeno-associated virus, retinal transduction, intravitreal injection, gene therapy

## Abstract

Optimizing adeno-associated virus (AAV) capsid and dosing selection is critical for the clinical translation of retinal gene therapy. This study aims to provide a comprehensive reference by comparing the transduction efficiency, cellular tropisms, and temporal retinal expression patterns of various AAV serotypes for intravitreal retinal gene therapy. A series of AAV vectors were intravitreally injected into C57BL/6J mice. Retinal tissues were harvested 4 weeks post-injection to evaluate the transgene expression and cellular tropisms by immunostaining. Both ssAAV2.NN and scAAV2.NN vectors at a dose escalation were administered, with similar assessments conducted at 2 and 4 weeks post-injection. Additionally, the early-phase retinal transduction profiles of AAV vectors were detected at multiple time points within 12 weeks following administration. Stronger green fluorescent protein (GFP) fluorescence was observed in retinas intravitreally transduced with AAV2.NN, AAV2.GL, and AAV8 vectors, with AAV2.GL showing greater co-localization with GS^+^ Müller cells and axons. Compared to ssAAV2.NN, scAAV2.NN vectors resulted in higher GFP fluorescence, despite similar transfected cell percentages (except for its increased co-labeling with Calbindin^+^ horizontal cells). A dose-dependent level of GFP fluorescence was noted in scAAV2.NN vectors, with greater co-labeling with Rbpms^+^ retinal ganglion cells at high doses. Interestingly, GFP fluorescence was detectable as early as 3 days post-injection in both ssAAV2.NN and scAAV2.NN vectors, with no prominent differences in the intensity until day 7. AAV2.GL vectors achieved higher transgene expression and broader cellular transduction via the intravitreal route. scAAV2.NN vectors presented stronger transgene expression in a dose-dependent manner. The transgene expression from both ssAAV2.NN and scAAV2.NN vectors can be detected as early as 3 days post-injection. Our study provides key insights for early-stage monitoring and vector and dosage selection in future clinical application for intravitreal gene therapy.

**IMPORTANCE:**

The retinal transduction efficiency and cellular tropisms of serial AAV serotypes, including AAV2, AAV2.7m8, AAV2.NN, AAV2.GL, AAV8, AAV11, and AAV.SPR, were simultaneously and unbiasedly quantified and compared following intravitreal injection. Transgene fluorescence was detectable in cells as early as 3 days post-injection in retinas intravitreally transduced with both ssAAV2.NN and scAAV2.NN vectors. The timeliness of the onset and level of transgene expression in retinas intravitreally transduced with ssAAV2.NN and scAAV2.NN vectors were characterized during the early phase post-injection. Differences in retinal transduction efficiency and cellular tropisms of scAAV2.NN vectors at varying doses via intravitreal injection are described.

## INTRODUCTION

Gene therapy for inherited retinal diseases has developed rapidly in the past decade, with dozens of clinical trials for disorders currently ongoing (1–3). Adeno-associated virus (AAV) has emerged as a potential gene delivery tool due to its low immunogenicity, high safety, inability to integrate in the body, and long-term wide expression in tissues (4–6). AAV-mediated gene therapy has shown substantial disease modifications in many monogenic disorders and even the possibility of completely curing the disease (4, 7, 8). It holds great promise, particularly in immune-privileged compartments, such as the central nervous system (CNS) and the retina (9, 10).

As a powerful vehicle for gene delivery, the development of novel-engineered AAV vectors has come to our attention. Over 10 different AAV serotypes have been utilized as vehicles for gene therapy in clinical trials (11). AAV2 has been indicated to remain the most widely used throughout the study period with the most evidence of safety and efficacy, validated in more than 40 completed clinical trials (12, 13). There have been a growing number of clinical trials applying AAV vectors for gene therapy on CNS disorders within the last decade (13–15). The broad-spectrum promoters, including CBA, CAG, and CMV, remain the three most commonly used as they have been reported to be effective and applicable to a wide range of systems in previous studies (13). The successful clinical application of AAV2 in the treatment of Leber congenital amaurosis became the first gene therapy (Luxturna), approved by the Food and Drug Administration, and has already been administered in the clinic (16, 17).

In preclinical studies, AAV2 can efficiently transduce the retinal pigment epithelium (RPE) and retinal ganglion cells (RGCs) via subretinal and intravitreal injection (18–20). However, its lack of ability to cross retinal neurons may limit its applications (19, 21). Targeting of retinal neurons other than RGCs still relies on subretinal delivery, which may lead to increased substantial risks of surgical complications, such as central retinal thinning and macular hole formation (22, 23). Not only that, the expertise and equipment required for subretinal injection will largely restrict its treatment availability (22, 24). Although alternative injection methods, including the sub-inner limiting membrane and suprachoroidal injection, could reduce potential risks of surgical complications, the intravitreal route, a simple and precise delivery method, is still more suitable for retinal gene therapy in the clinic (25–27). Hence, there is still an urgent need to search for novel AAV candidates to better target different retinal cell types via intravitreal injection, which holds great promise for clinical application.

Significant efforts have been undertaken by several research teams to develop novel-engineered AAV vectors for efficient retinal transduction. Notable examples of AAV variants including AAV2.7m8, AAV2.NN, and AAV2.GL have been selected and validated in mice, dogs, non-human primates, retinal organoids, and human retinal explants via intravitreal or subretinal delivery (20, 28–30). The subretinal injection of AAV.SPR exhibited high transduction efficiency and strong lateral diffusion ability in retinas in a preclinical study (31). AAV11 has been described as a potential vehicle that can efficiently and specifically realize transgene expression targeting bipolar cells (BCs) via intravitreal injection (http://epub.cnipa.gov.cn/Sw/SwDetail). These novel AAV vectors play an active role in expanding the clinical applicability of retinal gene therapy. However, a parallel comparison study has never been performed in intravitreal or subretinal delivery. The current study was to screen suitable AAV candidates for retinal gene therapy by comparing retinal transduction efficiency and cellular tropisms via intravitreal injection.

**Fig 1:**
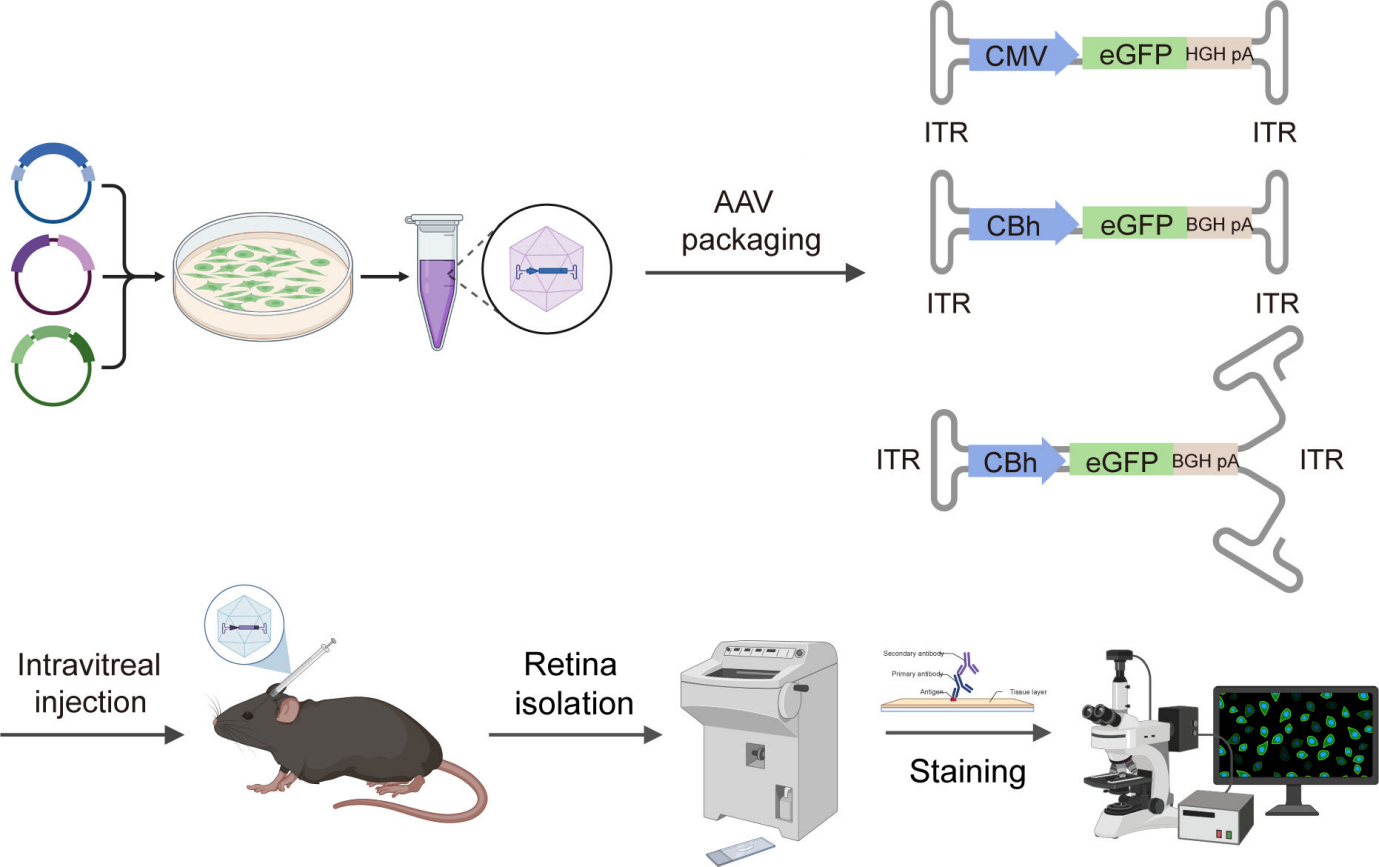
Schematic flowchart of experiments conducted in the study.

scAAV vectors have always been regarded as a highly efficient delivery tool for gene therapy due to their earlier onset and higher level of transgene expression compared to ssAAV (32–34). However, studies on their retinal transduction as well as the onset and level of transgene expression in the very early stage following intravitreal injection are still lacking. Moreover, the selection of appropriate administration dosages to reduce immune and inflammatory responses on the basis of achieving better therapeutic effects remains a tough problem in clinical gene therapy. The comparison was consequently performed among ssAAV and scAAV vectors at varying doses, with their retinal transduction profiles and the timeliness of transgene expression patterns discussed in this study at the same time. The flow chart is shown in Fig. 1.

## MATERIALS AND METHODS

### Animals

For this study, 6-week-old male C57BL/6J mice were procured from the Beijing Vital River Laboratory Animal Technology Co., Ltd. All steps were performed to minimize pain and discomfort to the animals and to reduce the number of animals required for the experiments.

### Preparation of AAV vectors

The recombinant AAV vectors used in this study were produced by using triple-plasmids transfected HEK293 cells as previously described (35), listed as follows in Table 1.

**TABLE 1:** Experimental design on AAV vectors, dosages, and sampling time^*a*^

AAV	Dosage (vg/eye)	Sampling time
ssAAV2.NN-CBh-eGFP-WPRE-BGHpA	4.00E+10	1 day p.i.
ssAAV2.NN-CBh-eGFP-WPRE-BGHpA	4.00E+10	3 days p.i.
ssAAV2.NN-CBh-eGFP-WPRE-BGHpA	4.00E+10	7 days p.i.
scAAV2.NN-CBh-eGFP-WPRE-BGHpA	4.00E+10	1 day p.i.
scAAV2.NN-CBh-eGFP-WPRE-BGHpA	4.00E+10	3 days p.i.
scAAV2.NN-CBh-eGFP-WPRE-BGHpA	4.00E+10	7 days p.i.
ssAAV2.NN-CBh-eGFP-WPRE-BGHpA	4.00E+10	2 weeks p.i.
scAAV2.NN-CBh-eGFP-WPRE-BGHpA	4.00E+10	2 weeks p.i.
scAAV2.NN-CBh-eGFP-WPRE-BGHpA	1.60E+09	4 weeks p.i.
scAAV2.NN-CBh-eGFP-WPRE-BGHpA	8.00E+09	4 weeks p.i.
scAAV2.NN-CBh-eGFP-WPRE-BGHpA	4.00E+10	4 weeks p.i.
ssAAV2-CMV-eGFP-WPRE-HGHpA	1.00E+09	4 weeks p.i.
ssAAV2.7m8-CMV-eGFP-WPRE-HGHpA	1.00E+09	4 weeks p.i.
ssAAV2.NN-CMV-eGFP-WPRE-HGHpA	1.00E+09	4 weeks p.i.
ssAAV2.GL-CMV-eGFP-WPRE-HGHpA	1.00E+09	4 weeks p.i.
ssAAV8-CMV-eGFP-WPRE-HGHpA	1.00E+09	4 weeks p.i.
ssAAV.SPR-CMV-eGFP-WPRE-HGHpA	1.00E+09	4 weeks p.i.
ssAAV11-CMV-eGFP-WPRE-HGHpA	1.00E+09	4 weeks p.i.

^*a*^ AAV: adeno-associated virus; BGHpA: bovine growth hormone poly A; CBh: cellobiohydrolase; CMV: cytomegalovirus; eGFP: enhanced green fluorescent protein; HGHpA: human growth hormone poly A; p.i.: post-injection; WPRE: woodchuck hepatitis virus posttranscriptional regulatory element.

### Intravitreal injection

Animals were anesthetized with 1% pentobarbital by intraperitoneal injection at a dose of 50 mg/kg, and topical oxybuprocaine hydrochloride (J20100128, Santen Pharmaceutical, Osaka, Japan) was applied for the cornea anesthesia to minimize any discomfort. Following the disinfection of skin around the eyes with iodophor, a 10 µL Hamilton Microsyringe (80365, Hamilton, Reno, NV, USA) with a 32-gauge needle (32G TipETW, Novo Nordisk A/S, Denmark) was applied for the injection under a dissecting microscope. Six-week-old C57BL/6J mice received 2 µL of AAV vectors via intravitreal injection into one eye.

The standardized injection procedure is as follows:

- A single, experienced laboratory technician performed all injections.
- The injection needle was held in place for 1 min post-injection (p.i.) to prevent reflux after gentle injection.
- The antibiotic eye ointment was topically applied after injection, and mice were placed on the heating pad until full awakening.
- Mice with iris hemorrhage, lens damage, leakage during puncture or after injection were excluded from the study.

### Immunofluorescence staining

The collected mouse eyes with a small hole pierced at the corneoscleral limbus were fixed in 4% paraformaldehyde (PFA, P6148-500G, Sigma) on ice for 30 mins, and complete eye cups were quickly dissected. After rinsing in phosphate buffered saline (PBS), eye cups were dehydrated with graded sucrose solutions (10%, 20%, and 30%) at 4°C for 1, 2, and 12 h, respectively. Eye cups were then embedded in optimal cutting temperature (OCT) compound (4583, SAKURA, USA) and stored at −80°C for freezing. Sagittal retinal cryosections were sliced passing through the optic nerve, with a thickness of 14 µm, by freezing microtome (G1066-1ML, Thermo Fisher, USA).

For immunofluorescence staining, retinal sections were first placed at room temperature for 30 min to 2 h before subsequent manipulations. Sections were washed in PBS and then blocked in PBS containing 2% bovine serum albumin (BSA) (4240GR100, Biofroxx, Germany) and 0.2% Triton X-100 (A7030, Amresco, USA) for 2 h at room temperature. The sections were subsequently incubated overnight at 4°C with specific primary antibodies diluted with PBS containing 1% BSA and 0.2% Triton X-100. After washing in PBS, the sections were incubated with diluted secondary antibodies for 2 h at room temperature in the dark. The sections were then washed in PBS and incubated with DAPI (1:100; D1306, Life Technologies, USA) for 10 min in the dark. Image collection and analyses were performed using the laser confocal microscope (LSM880, Zeiss, Germany) and ImageJ software, respectively. Primary and secondary antibodies used in this study were separately listed as follows in Tables 2 and 3.

**TABLE 2:** Primary antibodies used in the study^*a*^

Antibody	Species	Manufacturer	Dilution ratio	Labeled cells
Calbindin	Rabbit	SWANT	1:5,000	HCs
Chx10	Sheep	Thermo Fisher	1:20	BCs
Cone arrestin	Rabbit	Merck Millipore	1:500	Cones
PKCα	Rabbit	Santa Cruz	1:1,000	Rod-BCs
Pcp2	Goat	Santa Cruz	1:500	Rod-BCs and Cone-OFF BCs
Rbpms	Rabbit	Phosphosolutions	1:500	Ganglion cells
Recoverin	Rabbit	Merck Millipore	1:5,000	Photoreceptors
GS	Mouse	Merck Millipore	1:5,000	Müller cells
GFAP	Rabbit	DAKO	1:1,000	Astrocytes
GFP	Chicken	Abcam	1:1,000	N/A

^*a*^ GFAP: glial fibrillary acidic protein; GFP: green fluorescent protein; GS: glutamine synthetase; N/A: not available; Pcp2: Purkinje cell protein-2; PKCα: protein kinase cα; Rbpms: RNA binding protein with multiple splicin.

**TABLE 3:** Secondary antibodies used in the study

Antibody	Manufacturer	Dilution ratio
Alexa Fluor 647 AffiniPure Donkey Anti-Mouse IgG (H+L)	Jackson	1:500
Alexa Fluor 594 AffiniPure Donkey Anti-Rabbit IgG (H+L)	Jackson	1:500
Alexa Fluor 647 AffiniPure Donkey Anti-Rabbit IgG (H+L)	Jackson	1:500
Alexa Fluor 594 AffiniPure Donkey Anti-Goat IgG (H+L)	Jackson	1:500
Alexa Fluor 647 AffiniPure Donkey Anti-Sheep IgG (H+L)	Jackson	1:500
Alexa Fluor 488 AffiniPure Donkey Anti-Chicken IgG (H+L)	Jackson	1:500

As for the measurement of GFP fluorescence intensity, we have selected six regions of each retinal section under low magnification and separately photographed each under high magnification by confocal (LSM880, Zeiss, Germany), including tissues 250, 500, and 750 µm, respectively, from the optic nerve on the nasal and temporal side of retinas. The fluorescence intensity in each region has been measured using ImageJ, with average values recorded as the corresponding intensity of retinas.

### Hematoxylin-eosin (H&E) staining

The collected eye globes were fixed in 4% PFA for 24 h and dehydrated through a serial ethanol gradient (10009218, Sinopharm Chemical Reagent Co., Ltd.); specimens were immersed in xylene (10023428, Sinopharm Group Chemical Reagent Co., Ltd.) at 60°C for 10 to 15 min and paraffin-embedded. The paraffin blocks were cut sagittally with a thickness of 5 µm and counterstained with hematoxylin-eosin (H&E). An optical microscope (Axio Imager A2, Zeiss, Germany) and ImageJ software were used to respectively capture bright field images and measure the thickness of different retinal layers.

### ERG recordings

Electroretinogram (ERG) was performed using the RetiMINER IV electrophysiological system (IRC, Chongqing, China) to evaluate the retinal function. Mice were dark-adapted overnight, then intraperitoneally anesthetized with 1% pentobarbital at 50 mg/kg, and given oxybuprocaine hydrochloride and tropicamide (J20110007, Santen Pharmaceutical, Osaka, Japan) to respectively minimize ocular discomfort and dilute pupils. Mice were placed prone with both eyes fully symmetrically exposed to the light-giving device. Gold-wire electrodes were vertically placed on the cornea surface with a reference electrode inserted subcutaneously around the neck and a ground electrode with a bit of conductive paste at the end of the tail. Every single step was performed under dim red light throughout the scotopic recording. The full-field scotopic ERG responses were recorded at increasing light intensities from 0·0001 cd·s/m^2^ to 10·0 cd·s/m^2^. Each intensity was repeated at least once to confirm the reliability of recordings. Interstimulus intervals ranged from 5 to 60 s, depending on the stimulus intensity, between flash stimulations. A-wave and b-wave amplitudes were recorded and analyzed. After the experiment, antibiotic eye ointment was topically applied to reduce the risk of infection, and mice were placed on the heating pad until full awakening.

### Data analysis

GraphPad Prism 8.0 (GraphPad Software, Inc., San Diego, CA, USA) has been used for statistical analyses. Metering data tested by the Shapiro-Wilk test to fit the normal distribution is expressed as the mean ± standard deviation (SD) of the mean ($\overset{-}{X}$±SD). The homogeneity of variance has been assessed by the Brown-Forsythe test prior to the application of independent *t*-test for the comparison of two groups and one-way analysis of variance for the comparison of multiple groups. Sample sizes for each group were three or four. *P*-values less than 0.05 were considered statistically significant (^*^P＜0.05, ^**^P＜0.01, ^***^P＜0.001, ^****^P＜0.0001).

## RESULTS

### Retinal transduction profiles of serial AAV vectors via intravitreal injection

To evaluate the transduction efficiency and cellular tropisms of seven different AAV vectors including AAV2, AAV2.7m8, AAV2.NN, AAV2.GL, AAV8, AAV.SPR, and AAV11 via intravitreal injection, retinas were collected 4 weeks after administration and processed for immunofluorescence staining.

**Fig 2:**
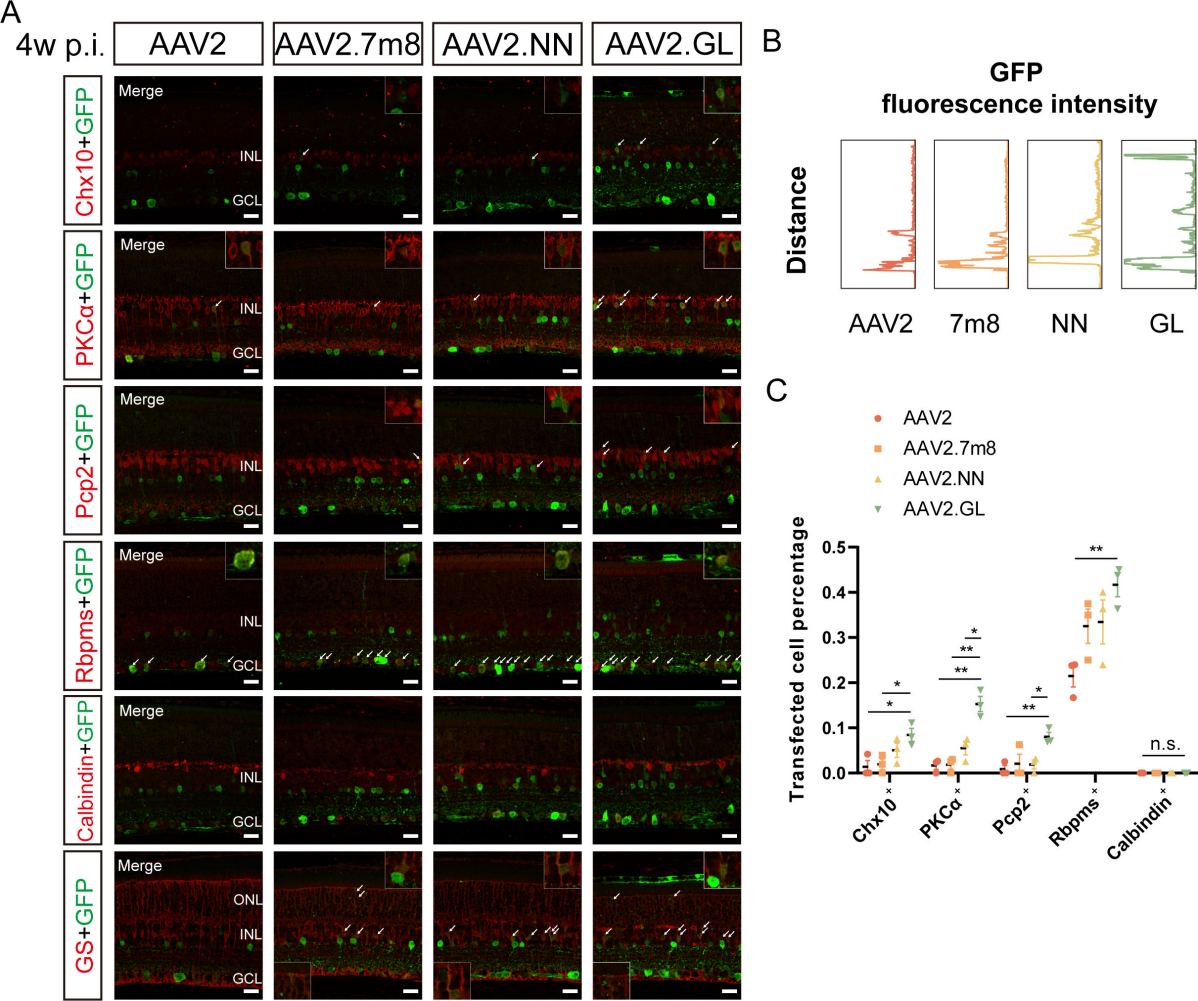
The comparison of retinal transduction profiles among AAV2 and three engineered AAV2 vectors including AAV2.7m8, AAV2.NN, and AAV2.GL 4 weeks after intravitreal injection. (**A**) The immunofluorescence staining of retinas transduced with AAV2 and three engineered AAV2 vectors 4 weeks after intravitreal injection. The green fluorescence is GFP; the red respectively refers to Chx10, PKCα, Pcp2, Rbpms, Calbindin, and GS, with a scale bar of 20 µm; and the images in the box were amplified and placed in the lower left and upper right corners. The white arrows indicate co-labeled parts. (**B**) The expression patterns of GFP fluorescence intensity in retinas transduced with AAV2 and three engineered AAV2 vectors 4 weeks after intravitreal injection. (**C**) The comparison of transfected percentages of Chx10^+^, PKCα^+^, Pcp2^+^, Rbpms^+^, and Calbindin^+^ cells among retinas transduced with AAV2 and three engineered AAV2 vectors 4 weeks after intravitreal injection. AAV2.GL vs. AAV2: Chx10^+^ ([0.085 ± 0.014] vs [0.014 ± 0.014], *P* < 0.05), PKCα^+^ ([0.153 ± 0.017] vs [0.017 ± 0.008], *P* < 0.01), Pcp2^+^ ([0.080 ± 0.010] vs [0.008 ± 0.008], *P* < 0.01), Rbpms^+^ ([0.471 ± 0.027] vs [0.215 ± 0.024], *P* < 0.01), AAV2.GL vs AAV2.7m8: Chx10^+^ ([0.085 ± 0.014] vs [0.019 ± 0.011], *P* < 0.05), PKCα^+^ ([0.153 ± 0.017] vs [0.018 ± 0.009], *P* < 0.01), Pcp2^+^ ([0.080 ± 0.010] vs [0.021 ± 0.021], *P* > 0.05), Rbpms^+^ ([0.471 ± 0.027] vs [0.325 ± 0.038], *P* > 0.05), AAV2.GL vs AAV2.NN: Chx10^+^ ([0.085 ± 0.014] vs [0.050 ± 0.016], *P* > 0.05), PKCα^+^ ([0.153 ± 0.017] vs [0.055 ± 0.015], *P* < 0.05), Pcp2^+^ ([0.080 ± 0.010] vs [0.019 ± 0.010], *P* < 0.05), Rbpms^+^ ([0.471 ± 0.027] vs [0.335 ± 0.048], *P* > 0.05). *P < 0.05, **P < 0.01, n.s., no significant differences, *n* = 3 in each group. AAV: adeno-associated virus; GFP: green fluorescent protein; PKCα: protein kinase cα; Pcp2: Purkinje cell protein-2; Rbpms: RNA binding protein with multiple splicing; GS: glutamine synthetase

**Fig 3:**
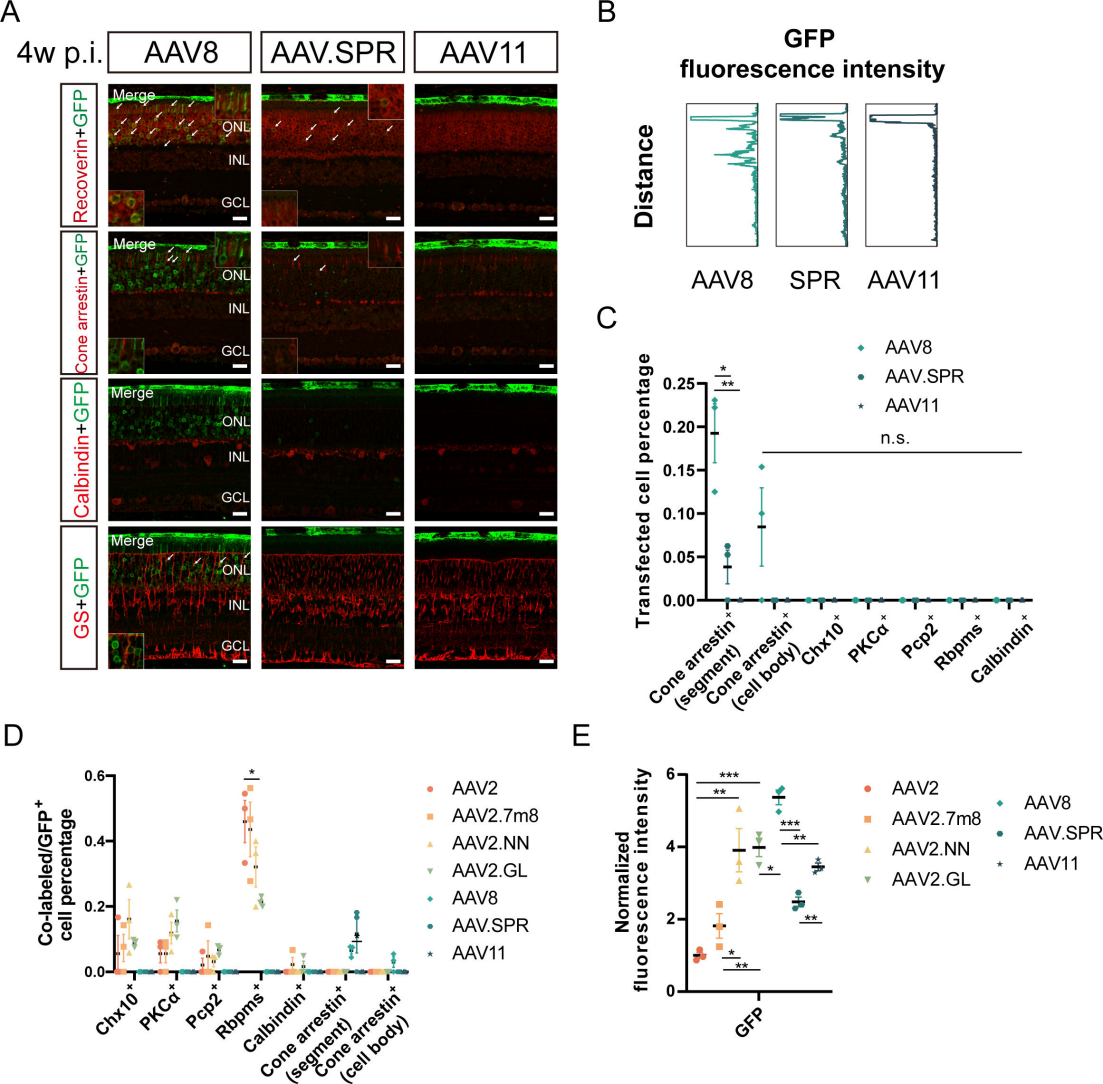
The comparison of retinal transduction profiles among serial AAV vectors including AAV2, AAV2.7m8, AAV2.NN, AAV2.GL, AAV8, AAV.SPR, and AAV11 4 weeks after intravitreal injection. (**A**) The immunofluorescence staining of retinas transduced with three different AAV vectors (AAV8, AAV.SPR, and AAV11) 4 weeks after intravitreal injection. The green fluorescence is GFP; the red respectively refers to Recoverin, Cone arrestin, Calbindin, and GS, with a scale bar of 20 µm; and the images in the box were amplified and placed in the lower left and upper right corners. The white arrows indicate co-labeled parts. (**B**) The expression patterns of GFP fluorescence intensity in retinas transduced with three different AAV vectors 4 weeks after intravitreal injection. (**C**) The comparison of transfected percentages of Cone arrestin^+^ segments, Cone arrestin^+^ cell bodies, Chx10^+^, PKCα^+^, Pcp2^+^, Rbpms^+^, and Calbindin^+^ cells among retinas transduced with three different AAV vectors 4 weeks after intravitreal injection. (**D**) The comparison of co-labeled/GFP^+^ cell percentages among retinas transduced with seven different AAV vectors (AAV2, AAV2.7m8, AAV2.NN, AAV2.GL, AAV8, AAV.SPR, and AAV11) 4 weeks after intravitreal injection. (**E**) The comparison of normalized GFP fluorescence intensity among retinas transduced with seven different AAV vectors 4 weeks after intravitreal injection. AAV2.GL vs. AAV2 ([3.988 ± 0.253] vs [1.000 ± 0.083], *P* < 0.001), AAV2.GL vs AAV2.7m8 ([3.988 ± 0.253] vs [1.818 ± 0.338], *P* < 0.01), AAV2. GL vs. AAV2.NN ([3.988 ± 0.253] vs [3.910 ± 0.596], *P* > 0.05), AAV2.GL vs AAV8 ([3.988 ± 0.253] vs [5.370 ± 0.201], *P* < 0.05), AAV2.GL vs AAV.SPR ([3.988 ± 0.253] vs [2.481 ± 0.132], *P* < 0.01), AAV2.GL vs AAV11 ([3.988 ± 0.253] vs [3.450 ± 0.106], *P* > 0.05), AAV2.NN vs AAV2 ([3.910 ± 0.596] vs [1.000 ± 0.083], *P* < 0.01), AAV2.NN vs AAV2.7m8 ([3.910 ± 0.596] vs [1.818 ± 0.338], *P* < 0.05), AAV2.NN vs AAV8 ([3.910 ± 0.596] vs [5.370 ± 0.201], *P* > 0.05), AAV2.NN vs AAV.SPR ([3.910 ± 0.596] vs [2.481 ± 0.132], *P* > 0.05), AAV2.NN vs AAV11 ([3.910 ± 0.596] vs [3.450 ± 0.106], *P* > 0.05). *P < 0.05, **P < 0.01, ***P < 0.001, n.s., no significant differences, *n* = 3 in each group. AAV: adeno-associated virus; GFP: green fluorescent protein; GS: glutamine synthetase; PKCα: protein kinase cα; Pcp2: Purkinje cell protein-2; Rbpms: RNA binding protein with multiple splicing

AAV2-injected retinas exhibited limited GFP fluorescence mainly in the ganglion cell layer (GCL) (Fig. 2A), indicative of high-level RGC transduction, with only sparse transfected cells in the inner nuclear layer (INL) (Fig. 2A; Fig. S1A). The relatively stronger GFP fluorescence could be observed in retinas transduced with AAV2.NN, AAV2.GL, AAV8, and AAV11 vectors (Fig. 2A, 3A and E; Fig. S1A). More transfected cells were detected in the INL and outer nuclear layer in AAV2.GL vectors (Fig. 2A; Fig. S1A), suggesting its certain ability to cross retinal neurons. This could also be demonstrated by the greater co-labeling with GS^+^ Müller cells and axons (shown by white arrows in Fig. 2A). However, AAV8, AAV.SPR, and AAV11 vectors favored the outer retinal layers via the intravitreal route, completely different from AAV2-based vectors (Fig. 3A; Fig. S1A). The expression patterns of GFP fluorescence intensity in retinas intravitreally transduced with seven different AAV vectors were depicted in Fig. 2B and 3B.

By statistics, the superiority of AAV2.GL over AAV2, AAV2.7m8, and AAV2.NN vectors in transfected percentages of Chx10^+^, PKCα^+^, Pcp2^+^ cells (BCs) and Rbpms^+^ cells (RGCs) was further confirmed (Fig. 3E, with the co-labeled parts shown by white arrows in Fig. 2A). Actually, AAV2.NN vectors also exhibited comparatively high transfected cell percentages, especially of Chx10^+^ cells, among AAV2-based vectors (Fig. 2C, with the co-labeled parts shown by white arrows in Fig. 2A). Among all transfected cells, the targeting of Rbpms^+^ cells was nearly up to 50% in AAV2 and AAV2.7m8 vectors, with almost 30% and 20%, respectively, in AAV2.NN and AAV2.GL vectors (Fig. 3D). The intravitreal delivery of AAV8 vectors resulted in higher transfected percentages of Cone arrestin^+^ segments (cone segments) ([0.193 ± 0.034] vs [0.038 ± 0.019], *P* < 0.05) ([0.193 ± 0.034] vs [0.000 ± 0.000], *P* < 0.01) and Cone arrestin^+^ cells (cone cell bodies) ([0.085 ± 0.045] vs [0.000 ± 0.000], *P* = 0.134) ([0.085 ± 0.045] vs [0.000 ± 0.000], *P* = 0.134) compared to AAV.SPR and AAV11 vectors (Fig. 3C, with the co-labeled parts shown by white arrows in Fig. 3A). No co-labeling with Chx10^+^, PKCα^+^, Pcp2^+^, and Rbpms^+^ cells was detected in retinas intravitreally transduced with these AAV vectors (AAV8, AAV.SPR, and AAV11) (Fig. 3A; Fig. S2A).

In conclusion, three engineered AAV2 vectors (AAV2.7m8, AAV2.NN, and AAV2.GL) outperformed AAV2, with the strongest transgene fluorescence detected in AAV2.GL, suggesting its higher transduction efficiency. Their transfected percentages of RGCs were obviously higher than that of BCs (Fig. 2C), conforming to the characteristics of AAV2 by intravitreal injection despite capsid modifications. However, AAV8, AAV.SPR, and AAV11 vectors were inclined to transfect the outer retinal layers via the intravitreal route, with the most prominent and unexpected performance by AAV8.

### Comparison of retinal transduction profiles between ssAAV2.NN and scAAV2.NN vectors via intravitreal injection

scAAV vectors have been generally considered to be more efficient in gene delivery compared to ssAAV. We, therefore, aim to further evaluate the transduction efficiency and cellular tropisms of ssAAV2.NN and scAAV2.NN vectors via intravitreal injection. Similarly, retinas were collected 2 weeks after administration and processed for immunofluorescence staining.

**Fig 4:**
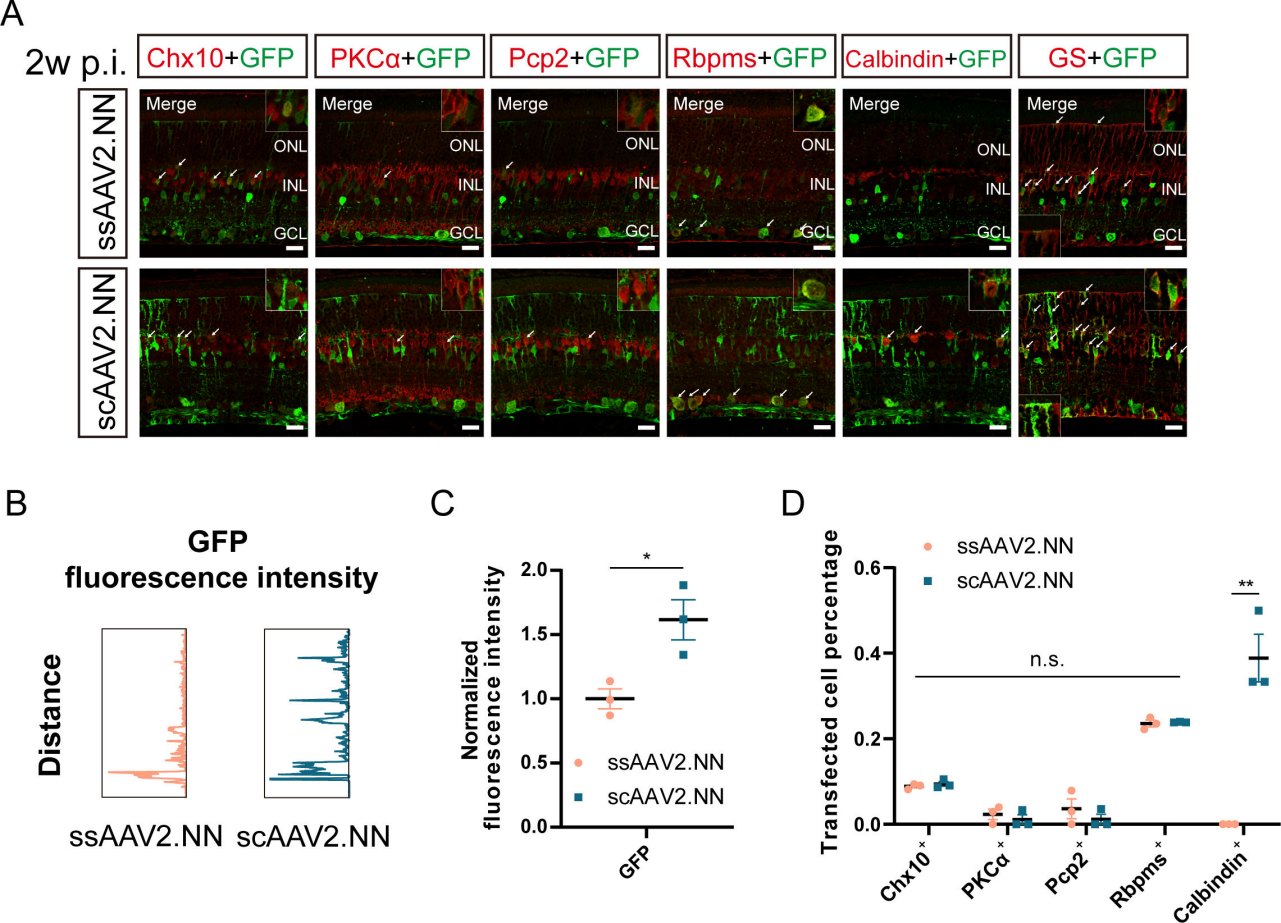
The comparison of retinal transduction profiles between ssAAV2.NN and scAAV2.NN vectors 2 weeks after intravitreal injection. (**A**) The immunofluorescence staining of retinas transduced with ssAAV2.NN and scAAV2.NN vectors 2 weeks after intravitreal injection. The green fluorescence is GFP; the red respectively refers to Chx10, PKCα, Pcp2, Rbpms, Calbindin, and GS, with a scale bar of 20 µm; and the images in the box were amplified and placed in the lower left and upper right corners. The white arrows indicate co-labeled parts. (**B**) The expression patterns of GFP fluorescence intensity in retinas transduced with ssAAV2.NN and scAAV2.NN vectors 2 weeks after intravitreal injection. (**C**) The comparison of normalized GFP fluorescence intensity between retinas transduced with ssAAV2.NN and scAAV2.NN 2 weeks after intravitreal injection. (**D**) The comparison of transfected percentages of Chx10^+^, PKCα^+^, Pcp2^+^, Rbpms^+^, and Calbindin^+^ cells between retinas transduced with ssAAV2.NN and scAAV2.NN 2 weeks after intravitreal injection. *P < 0.05, **P < 0.01, n.s., no significant differences, *n* = 3 in each group. AAV: adeno-associated virus; GFP: green fluorescent protein; PKCα: protein kinase C alpha; Pcp2: Purkinje cell protein-2; Rbpms: RNA binding protein with multiple splicing; GS: glutamine synthetase

Significantly stronger GFP fluorescence was detected in retinas transduced with scAAV2.NN vectors relative to ssAAV2.NN ([1.165 ± 0.156] vs [1.000 ± 0.007], *P* < 0.05) (Fig. 4A and C; Fig. S1B). Specifically, ssAAV2.NN-injected retinas exhibited GFP fluorescence mainly in the GCL and INL, while it was observed almost throughout all retinal layers in scAAV2.NN vectors (Fig. 4A; Fig. S1B). The greater co-labeling with Recoverin^+^ cells (photoreceptors) and GS^+^ cells and axons was also observed in retinas transduced with scAAV2.NN (shown by white arrows in Fig. 4A; Fig. S2B). These strongly supported the ability of scAAV2.NN vectors in mediating more efficient transduction across retinal neurons in a short time, although its single-stranded form was more inclined to transduce the inner retinal layers. The expression patterns of GFP fluorescence intensity in retinas intravitreally transduced with ssAAV2.NN and scAAV2.NN vectors were depicted in Fig. 4B.

However, no significant differences were detected in their transfected percentages of Chx10^+^, PKCα^+^, Pcp2^+^, and Rbpms^+^ cells. (Fig. 4D, with the co-labeled parts shown by white arrows in Fig. 4A). While retinas transduced with scAAV2.NN vectors showed a remarkably higher transfected percentage of Calbindin^+^ cells (horizontal cells, HCs) ([0.389 ± 0.056] vs [0.000 ± 0.000], *P* < 0.01) in comparison with ssAAV2.NN (Fig. 4D, with the co-labeled parts shown by white arrows in Fig. 4A).

It indicates that scAAV2.NN vectors may be more likely to transduce retinal cells other than RGCs and BCs, such as HCs and Müller cells, as shown by the higher transfected percentage of Calbindin^+^ cells and transgene fluorescence expressed along axons across the retina (Fig. 4A and D; Fig. S1B). Similarly, the transfected percentage of RGCs was higher than that of BCs in both ssAAV2.NN and scAAV2.NN vectors (Fig. 4D).

### Comparison of retinal transduction profiles among scAAV2.NN vectors at varying doses via intravitreal injection

In view of the significance of the dose-dependent transfection effect in clinical gene therapy, retinas intravitreally transduced with scAAV2.NN vectors at a five-fold dose gradient were collected 4 weeks after administration for immunofluorescence staining.

**Fig 5:**
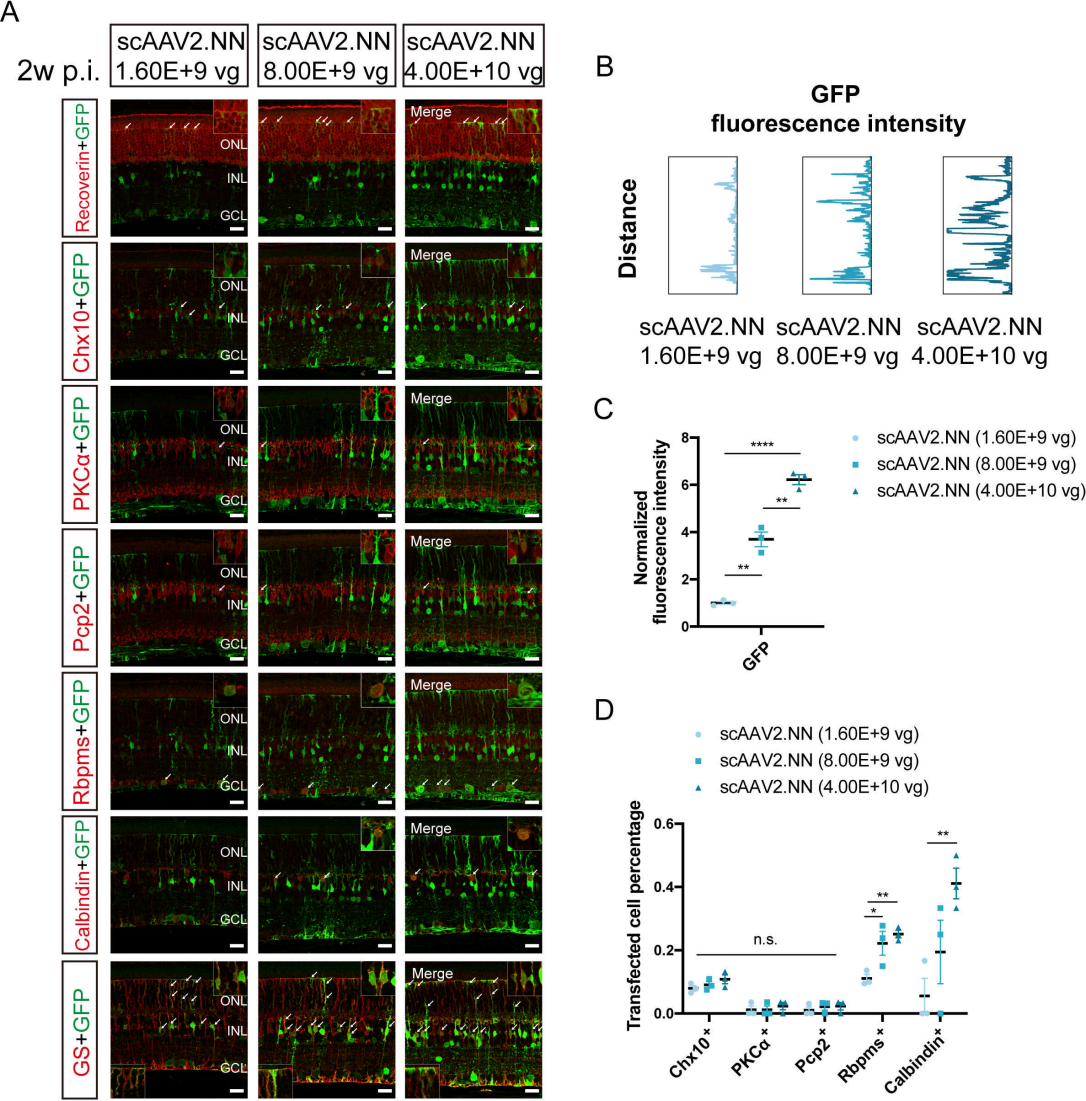
The comparison of retinal transduction profiles among scAAV2.NN vectors at a five-fold dose gradient 4 weeks after intravitreal injection. (**A**) The immunofluorescence staining of retinas transduced with scAAV2.NN vectors at a dose gradient 4 weeks after intravitreal injection. The green fluorescence is GFP; the red respectively refers to Recoverin, Chx10, PKCα, Pcp2, Rbpms, Calbindin, and GS, with a scale bar of 20 µm; and the images in the box were amplified and placed in the lower left and upper right corners. The white arrows indicate co-labeled parts. (**B**) The expression patterns of GFP fluorescence intensity in retinas transduced with scAAV2.NN vectors at a dose gradient 4 weeks after intravitreal injection. (**C**) The comparison of normalized GFP fluorescence intensity among retinas transduced with scAAV2.NN vectors at a dose gradient 4 weeks after intravitreal injection. (**D**) The comparison of transfected percentages of Chx10^+^, PKCα^+^, Pcp2^+^, Rbpms^+^, and Calbindin^+^ cells among retinas transduced with scAAV2.NN vectors at a dose gradient 4 weeks after intravitreal injection. Stock solution vs 5 × solution: Rbpms^+^ ([0.251 ± 0.012] vs [0.091 ± 0.010], *P* > 0.05), Calbindin^+^ ([0.411 ± 0.048] vs [0.194 ± 0.100], *P* > 0.05); stock solution vs 25 × solution: Rbpms^+^ ([0.251 ± 0.012] vs [0.080 ± 0.009], *P* < 0.01), Calbindin^+^ ([0.411 ± 0.048] vs [0.056 ± 0.056], *P* < 0.01); 5 × solution vs 25 × solution: Rbpms^+^ ([0.091 ± 0.010] vs [0.080 ± 0.009], *P* < 0.05), and Calbindin^+^ ([0.194 ± 0.100] vs [0.056 ± 0.056], *P* > 0.05). *P < 0.05, **P < 0.01, ****P < 0.0001, n.s., no significant differences, *n* = 3 in each group. AAV: adeno-associated virus; GFP: green fluorescent protein; PKCα: protein kinase C alpha; Pcp2: Purkinje cell protein-2; Rbpms: RNA binding protein with multiple splicing; GS: glutamine synthetase

The strongest GFP fluorescence could be obviously detected in the stock solution group ([6.221 ± 0.209] vs [3.694 ± 0.309], *P* < 0.01) ([6.221 ± 0.209] vs [1.000 ± 0.066], *P* < 0.0001), with the intensity declining following descending dosages (Fig. 5A and C; Fig. S1C). Retinas transduced with scAAV2.NN vectors at three doses showed GFP fluorescence almost throughout all retinal layers (Fig. 5A; Fig. S1C), with great co-labeling with Recoverin^+^ cells and GS^+^ cells and axons (shown by white arrows in Fig. 5A). It further demonstrated the higher transduction efficiency of scAAV2.NN vectors across the retina even at a relatively low dose via intravitreal injection. The expression patterns of GFP fluorescence intensity in retinas intravitreally transduced with scAAV2.NN vectors at a fivefold dose gradient were depicted in Fig. 5B.

Surprisingly, no distinct differences in transfected percentages of Chx10^+^, PKCα^+^, and Pcp2^+^ cells were perceived among three groups, except for Rbpms^+^ and Calbindin^+^ cells (Fig. 5D, with the co-labeled parts shown by white arrows in Fig. 5A). This suggested that the intravitreal injection of scAAV2.NN vectors within 25× dilution would not change their transduction properties for BCs. However, the stock solution group and the 5× dilution group exhibited slightly higher transfected percentages of Rbpms^+^ and Calbindin^+^ cells compared to the 25× dilution group (Fig. 5D, with the co-labeled parts shown by white arrows in Fig. 5A), indicating that 25× dilution would mainly weaken its transduction characteristics for RGCs and HCs.

The transfected percentages of RGCs, in the same way, were higher than that of BCs in scAAV2.NN vectors at three doses (Fig. 5D). That is, the intravitreal delivery of scAAV2.NN vectors within 25× dilution will not change its transduction preference for RGCs. At the same time, scAAV2.NN vectors showed efficient transduction ability to cross the retina via the intravitreal route within 25× dilution, reflected by the transgene fluorescence expressed throughout all retinal layers (Fig. 5A; Fig. S1C).

### Comparison of retinal transduction profiles between ssAAV2.NN and scAAV2.NN vectors in the early stage after intravitreal injection

To explore the onset and level of transgene expression in the very early stage after intravitreal delivery of AAV vectors, retinas transduced with ssAAV2.NN and scAAV2.NN vectors were collected respectively at 1, 3, and 7 days p.i. and processed for immunofluorescence staining.

**Fig 6:**
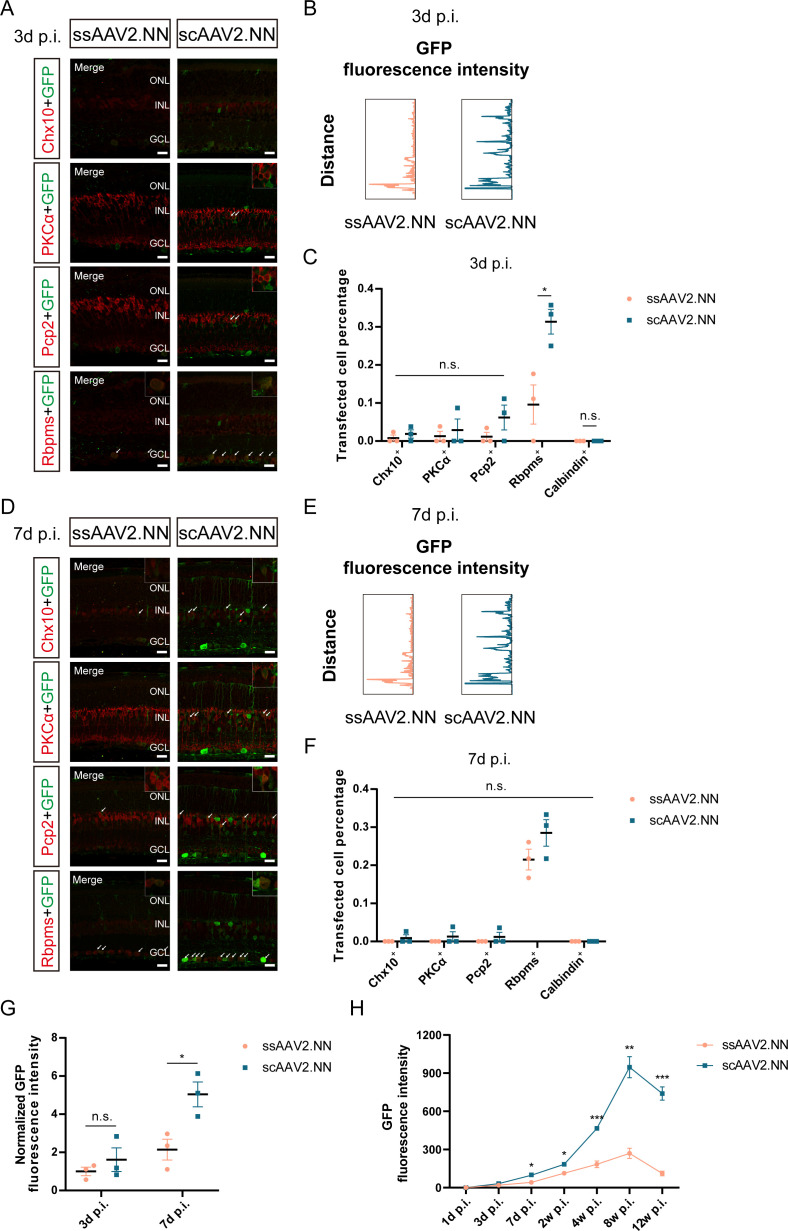
The comparison of retinal transduction profiles between ssAAV2.NN and scAAV2.NN vectors in the early stage after intravitreal injection. (**A**) The immunofluorescence staining of retinas transduced with ssAAV2.NN and scAAV2.NN vectors at 3 days after intravitreal injection. The green fluorescence is GFP; the red respectively refers to Chx10, PKCα, Pcp2, and Rbpms, with a scale bar of 20 µm; and the images in the box were amplified and placed in the upper right corners. The white arrows indicate co-labeled cells. (**B**) The expression patterns of GFP fluorescence intensity in retinas transduced with ssAAV2.NN and scAAV2.NN vectors at 3 days after intravitreal injection. (**C**) The comparison of transfected percentages of Chx10^+^, PKCα^+^, Pcp2^+^, and Rbpms^+^ cells between retinas transduced with ssAAV2.NN and scAAV2.NN vectors at 3 days after intravitreal injection. (**D**) The immunofluorescence staining of retinas transduced with ssAAV2.NN and scAAV2.NN vectors at 7 days after intravitreal injection. The green fluorescence is GFP; the red respectively refers to Chx10, PKCα, Pcp2, and Rbpms, with a scale bar of 20 µm; and the images in the box were amplified and placed in the upper right corners. The white arrows indicate co-labeled cells. (**E**) The expression patterns of GFP fluorescence intensity in retinas transduced with ssAAV2.NN and scAAV2.NN vectors at 7 days after intravitreal injection. (**F**) The comparison of transfected percentages of Chx10^+^, PKCα^+^, Pcp2^+^, and Rbpms^+^ cells between retinas transduced with ssAAV2.NN and scAAV2.NN vectors at 7 days after intravitreal injection. (**G**) The comparison of normalized GFP fluorescence intensity between retinas transduced with ssAAV2.NN and scAAV2.NN vectors at 3 and 7 days after intravitreal injection. (**H**) The changes in GFP fluorescence intensity of retinas transduced with ssAAV2.NN and scAAV2.NN vectors at 1, 3, and 7 days and 2, 4, 8, and 12 weeks after intravitreal injection. *P < 0.05, n.s., no significant differences, *n* = 3 in each group. AAV: adeno-associated virus; GFP: green fluorescent protein; PKCα: protein kinase cα; Pcp2: Purkinje cell protein-2; Rbpms: RNA binding protein with multiple splicing; GS: glutamine synthetase

The GFP fluorescence could be detected as early as 3 days p.i. in both ssAAV2.NN and scAAV2.NN vectors (Fig. 6A; Fig. S1D), with no significant differences in the intensity (Fig. 6G) and transfected percentages of Chx10^+^, PKCα^+^, and Pcp2^+^, except for Rbpms^+^ ([0.096 ± 0.052] vs [0.313 ± 0.032], *P* < 0.05) cells (Fig. 6C, with the co-labeled parts shown by white arrows in Fig. 6A). It pointed out that the retinal transduction efficiency between ssAAV2.NN and scAAV2.NN vectors exhibited no obvious differences in the very early stage after administration, apart from preferences for RGCs.

An increased GFP fluorescence could be observed at 7 days p.i. (Fig. 6D; Fig. S1D), with prominently higher intensity in scAAV2.NN-injected retinas ([5.041 ± 0.649] vs [2.144 ± 0.548], *P* < 0.05) (Fig. 6G). However, no significant differences in the transfected percentage of Rbpms^+^ cells between ssAAV2.NN and scAAV2.NN vectors were revealed (Fig. 6F, with the co-labeled parts shown by white arrows in Fig. 6D). The results demonstrated that differences in the transduction of RGCs only existed in the very early stage after intravitreal injection. The expression patterns of GFP fluorescence intensity in retinas intravitreally transduced with ssAAV2.NN and scAAV2.NN vectors at different time points were respectively depicted in Fig. 6B and E, with the timeline of GFP fluorescence expression in the early phase following administration plotted in Fig. 6H, which continued to increase within 8 weeks p.i. and gradually decreased later on.

On the whole, scAAV2.NN vectors exhibited an earlier onset and higher level of transgene expression in the early stage after intravitreal injection. The fluorescence could be detected in the inner and outer retinal layers, especially GCL, in retinas transduced with scAAV2.NN vectors, which was limited to GCL in ssAAV2.NN (Fig. 6A and D; Fig. S1D). It also suggests that ssAAV2.NN and scAAV2.NN vectors both show preferences for the prior transduction of RGCs in the very early stage following intravitreal administration.

## DISCUSSION

Three novel-engineered AAV2 vectors, including AAV2.7m8, AAV2.NN, and AAV2.GL, have been previously screened as potentially powerful tools for retinal gene therapy (20, 29, 30). In the current study, a series of AAV vectors were intravitreally injected into C57BL/6J mice to further explore their retinal transduction profiles. All AAV2-based vectors showed transduction largely restricted to the inner retinal layer, partly different from the research of Pavlou et al. (20). The differences may be mainly due to the lower administration dosages and single-stranded AAV vectors applied in our study. They all exhibited preferences for the prior transduction of RGCs, which could also be detected in AAV2.NN vectors in the very early stage (3 days p.i. and 7d p.i.) after intravitreal delivery, since RGCs are always first affected via intravitreal injection diffusion. However, the proportion of targeted RGCs among all transfected cells in AAV2.GL vectors is about only 20%, indicating that many other retinal cells, including HCs and Müller cells, were probably transfected as well.

In accordance with the study by Pavlou et al. (20), AAV2.GL achieved the strongest GFP fluorescence among three engineered AAV2 vectors in our study. Likewise, scAAV vectors resulted in transgene fluorescence spanned throughout all retinal layers following intravitreal injection in both studies. The efficient retinal transduction with scAAV2.NN and scAAV2.GL vectors via intravitreal delivery in non-human primates was also proved in that study, reflected by the high transfected percentage of photoreceptors in the fovea, with a similar phenomenon observed in human retinal explants (20). The differences from what has been observed in C57BL/6J mice may be due to differences between species.

However, the transgene fluorescence was detected in the inner retinal layer in the intravitreal group and in photoreceptors and RPE in the subretinal group a year after AAV8 injection in the study by Igarashi et al. (36), different from our study. Although broad-spectrum promoters (CMV and CAG) were utilized in both studies, the limited dosage in our study might lead to the differences in their retinal transduction profiles. However, the underlying causes and mechanisms still need to be further explored. The higher transduction efficiency of the outer retinal layer with AAV8 relative to AAV.SPR-, AAV11-, and AAV2-based vectors via the intravitreal route in our study may facilitate the treatment of retinal degeneration mainly with photoreceptor defects. Similar properties of AAV8 via subretinal delivery have been validated in another study (32). Unexpectedly, the intravitreal injection of AAV.SPR and AAV11 resulted in transgene fluorescence only limited to RPE in the current study, inconsistent with previous research (http://epub.cnipa.gov.cn/Sw/SwDetail) (31). The discrepancies may be attributed to different administration dosages and routes and promoters applied.

The earlier onset and higher level of transgene expression in retinas intravitreally transduced with scAAV vectors have been validated in previous research (32–34). As we all know, one of the rate-limiting steps for the onset of transgene expression is the conversion of the single-stranded DNA vector genome into the double-stranded, which can be bypassed by scAAV vectors (34, 37). However, no significant differences in transfected percentages of BCs and RGCs were revealed between ssAAV2.NN and scAAV2.NN vectors at 2 weeks p.i. in our study, while scAAV2.NN showed greater co-labeling with HCs.

The transduction of RPE and photoreceptors following subretinal delivery of AAV8 was respectively visible as early as 24 h and 5 days p.i. in the study by Natkunarajah et al. (32). However, dose-matched AAV2 and AAV5 vectors did not result in observable fluorescence until 1–2 weeks after administration. According to the findings by Yokoi et al. (33), the transgene fluorescence could be observed limited to RPE at 3 days following subretinal injection of both ssAAV2 and scAAV2 vectors in the high-dose group, which was detected in retinal cells at 7 days in scAAV2 and 28 days in ssAAV2. A study by Bennett et al. (38) showed that the fluorescence was first detectable 7 days after the subretinal injection of ssAAV vectors in the ophthalmoscopic observation. These results differ from the traditional concept that the transgene expression is usually detectable 2–3 weeks after subretinal delivery of ssAAV vectors (38, 39).

Although similar circumstances have been presumed to occur in the intravitreal injection of AAV vectors, studies on its early onset of transgene expression in retinal gene therapy are deficient. In the current study, the transgene fluorescence was visible as early as 3 days p.i. in both ssAAV2.NN and scAAV2.NN vectors, with no significant differences in the intensity. It suggests the similar onset of transgene expression of single- and double-stranded AAV vectors via intravitreal injection. Even more surprising, the transgene fluorescence in retinal cells (RGCs) could also be observed as early as 3 days p.i. in both of them in our study. The higher transfected percentage of RGCs in scAAV2.NN vectors in the very early stage (3 days p.i.) following administration can help us to recognize great advantages of scAAV vectors in the treatment of rapidly progressing retinal dystrophies characterized by RGC degeneration. Considering that the novel phenomenon might be attributed to the greater transduction efficiency of engineered AAV vectors, ssAAV2 and scAAV2 were further explored. Analogously, the intravitreal injection of ssAAV2 and scAAV2 vectors resulted in detectable transgene fluorescence in retinal cells (RGCs) as early as 3 days p.i. (Fig. S1D). The peak of transgene expression occurred at 8 weeks p.i. in both ssAAV2.NN and scAAV2.NN vectors in our study, consistent with the peak time of 6 to 8 weeks after subretinal delivery of ssAAV vectors reported by prior studies (38, 39).

Nevertheless, it has been reported that the transgene expression achieved by scAAV vectors via intramuscular injection may increase harmful immune responses (40). However, the extent to which this can be analogous to the intravitreal injection is unknown. According to clinical outcomes on retinal gene therapy (https://clinicaltrials.gov/), all AAV-based gene therapy products virtually exhibited safety profiles when administered by intravitreal injection, whether it is single- or double-stranded. Although transient immune inflammatory responses have been observed in very few participants, they can be gradually eliminated by the application of steroids (9, 13, 41). It was further verified in the current study that the retinal layers remained intact and regular in arrangement with normal thickness in those intravitreally transduced with ssAAV2.NN and scAAV2.NN vectors at varying doses (Fig. S3A and B), while stronger GFAP fluorescence could be detected in scAAV2.NN vectors when compared to ssAAV2.NN (Fig. S3C and D). Similarly, the stock solution group of scAAV2.NN vectors exhibited significantly stronger GFAP fluorescence at 4 weeks p.i., with 5× and 25× dilution groups showing no obvious differences compared to the normal (Fig. S3C and D). However, since the sampling was performed shortly after administration, we expected that the higher-level expression of retinal inflammatory protein detected in scAAV2.NN vectors would gradually fade over time. No significant changes were observed in a-wave and b-wave amplitudes under the scotopic condition among C57BL/6J mice intravitreally injected with buffer solution, ssAAV2.NN, and scAAV2.NN vectors at varying doses (Fig. S4A and B), indicating no adverse effects on the visual function. Of course, the selection of appropriate AAV candidates and administration dosages in clinical gene therapy is still not negligible, so as to reduce or even avoid the occurrence of immune and inflammatory responses as much as possible, presumably of substantial benefits for rapidly progressing retinal dystrophies.

Taken together, our work explored the retinal transduction profiles of seven different AAV vectors including AAV2, AAV2.7m8, AAV2.NN, AAV2.GL, AAV8, AAV.SPR, and AAV11 on the basis of previous research. The comparison among ssAAV2.NN and scAAV2.NN vectors at varying doses was also carried out, with the very early onset of transgene expression detected at the same time. Although the subretinal delivery is more widely used in the treatment of retinal degeneration for the purpose of photoreceptor transduction, the intravitreal administration, as a non-invasive delivery method, has been considered more efficient in penetrating retinas (22, 33). Based on these concomitant advantages, the exploration of AAV-mediated intravitreal gene therapy that can achieve efficient and specific retinal transduction should be encouraged. Our study may provide a reference in the selection of appropriate AAV candidates and administration dosages for intravitreal gene therapy. Besides, the very early onset of transgene expression and retinal transduction profiles of ssAAV2.NN and scAAV2.NN vectors in the early stage following administration are interesting and unexpected.

However, the limitations of our study are listed as follows: (i) the potential of novel AAV vectors can be further explored in different species, including non-human primates and human retinal explants, since there usually exist interspecies variations in their retinal transduction profiles; (ii) promoters specifically targeting different retinal cell types, such as Rho, GRK1, and TRPM1, can also be utilized in future translational studies; (iii) the comparison of the timing of the plateau of transgene expression and the expression level during the plateau in retinas transduced with ssAAV and scAAV vectors is still lacking and currently in progress; (iv) the inner limiting membrane has been identified as a barrier for intravitreal gene therapy. Pronase E and ICG-mediated photodisruption may considerably improve the transduction efficiency (42, 43). T-cell protein tyrosine phosphatase and protein phosphatase 5 can also serve to improve the retinal transduction efficiency (44–46).

Overall, bringing safer and more effective gene therapy to future clinical practice is our common goal. More transitional studies are needed, and there is still a long way to go.

## Data Availability

The authors confirm that the data of this study are available in the article and supplemental material. Detailed values for all data points shown in graphs are presented in supplemental Data Set S1. Any further information is also available per request.

## SUPPLEMENTAL MATERIAL

Data Set S1 jvi.00637-25-s0001.xlsx Detailed values for all data points shown in graphs.

Supplemental figures jvi.00637-25-s0002.docx Figures S1 to S4.

ASM does not own the copyrights to Supplemental Material that may be linked to, or accessed through, an article. The authors have granted ASM a non-exclusive, world-wide license to publish the Supplemental Material files. Please contact the corresponding author directly for reuse.
