# Supplementary material for: Retinal transduction profiling of diverse AAV serotypes via intravitreal injection
Source: J Virol. 2025 Aug 12;99(9):e00637-25. doi: 10.1128/jvi.00637-25 (PMC12455963; doi:10.1128/jvi.00637-25)
Supplement: Supplemental figures — Figures S1 to S4. [file jvi.00637-25-s0002.docx]

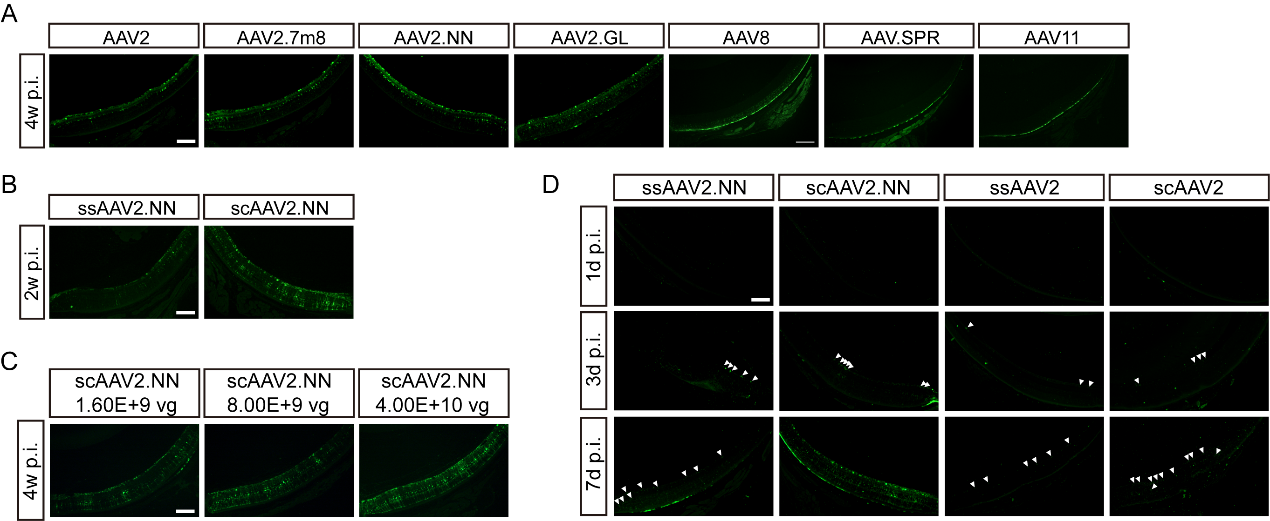


**Figure S1. The comparison of transgene fluorescence in retinas transduced with seven different AAV vectors including AAV2, AAV2.7m8, AAV2.NN, AAV2.GL, AAV8, AAV.SPR, and AAV11 4 weeks after intravitreal injection; with ssAAV2.NN and scAAV2.NN vectors 2 weeks after intravitreal injection; with scAAV2.NN vectors at a dose gradient 4 weeks after intravitreal injection; with ssAAV2.NN and scAAV2.NN vectors at 1, 3, and 7 days after intravitreal injection.** (**A**) The immunofluorescence staining of retinas transduced with seven different AAV vectors 4 weeks after intravitreal injection. The green fluorescence refers to GFP, with a scale bar of 200 μm. (**B**) The immunofluorescence staining of retinas transduced with ssAAV2.NN and scAAV2.NN vectors 2 weeks after intravitreal injection. The green fluorescence refers to GFP, with a scale bar of 200 μm. (**C**) The immunofluorescence staining of retinas transduced with scAAV2.NN vectors at a dose gradient 4 weeks after intravitreal injection. The green fluorescence refers to GFP, with a scale bar of 200 μm. (**D**) The immunofluorescence staining of retinas transduced with ssAAV2.NN and scAAV2.NN vectors at 1, 3, and 7 days after intravitreal injection. The green fluorescence refers to GFP, with a scale bar of 200 μm. AAV: adeno-associated virus; GFP: green fluorescent protein


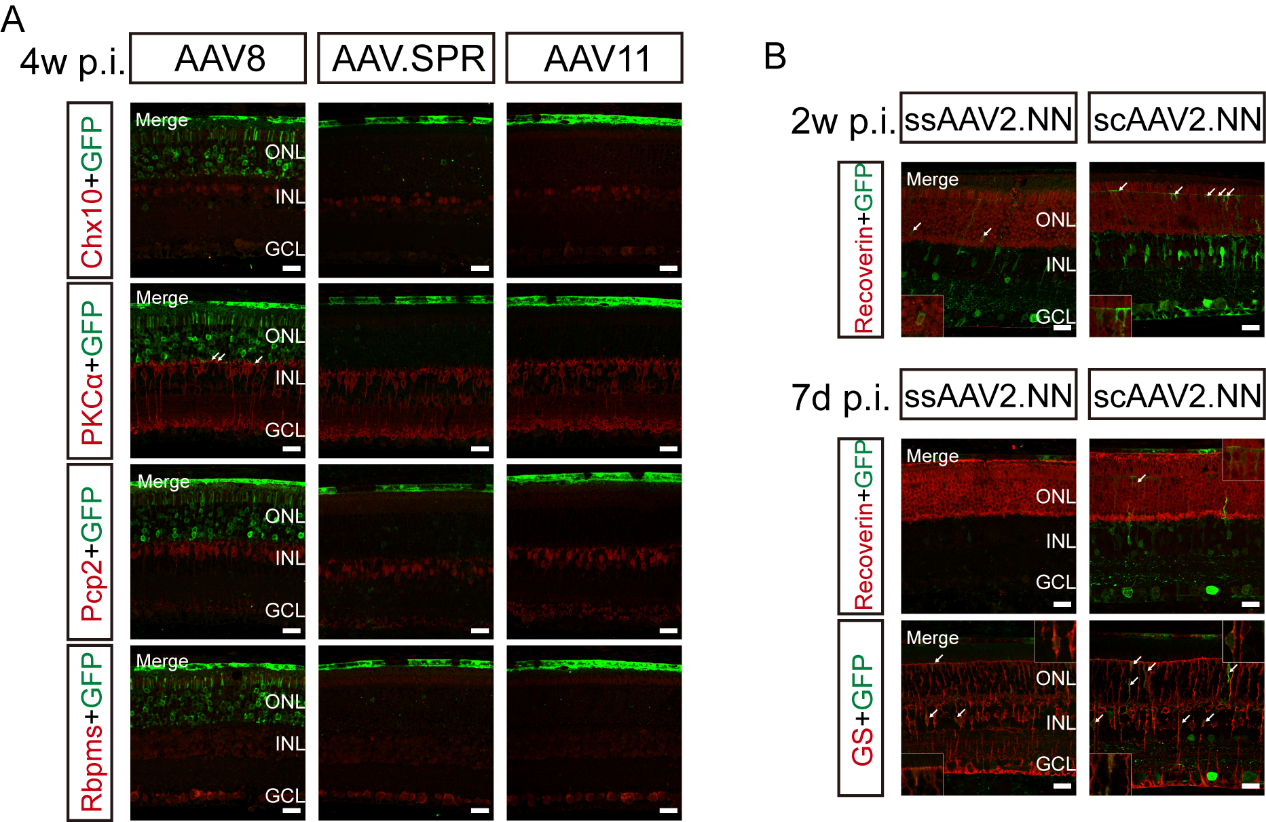


**Figure S2. The comparison of retinal transduction profiles of three different AAV vectors including AAV8, AAV.SPR, and AAV11 4 weeks after intravitreal injection; with** **ssAAV2.NN and scAAV2.NN vectors at 7 days and 2 weeks after intravitreal injection.** (**A**) The immunofluorescence staining of retinas transduced with three different AAV vectors 4 weeks after intravitreal injection. The green fluorescence is GFP, the red respectively refers to Chx10, PKCα, Pcp2, and Rbpms, with a scale bar of 20 μm. The white arrows indicate co-labeled parts. (**B**) The immunofluorescence staining of retinas transduced with ssAAV2.NN and scAAV2.NN vectors at 7 days and 2 weeks after intravitreal injection. The green fluorescence is GFP, the red respectively refers to Recoverin and GS, with a scale bar of 20 μm, and the images in the box were amplified and placed in the lower left and upper right corners. The white arrows indicate co-labeled parts. AAV: adeno-associated virus; GFP: green fluorescent protein; PKCα: protein kinase cα; Pcp2: purkinje cell protein-2; Rbpms: RNA binding protein with multiple splicing; GS: glutamine synthetase


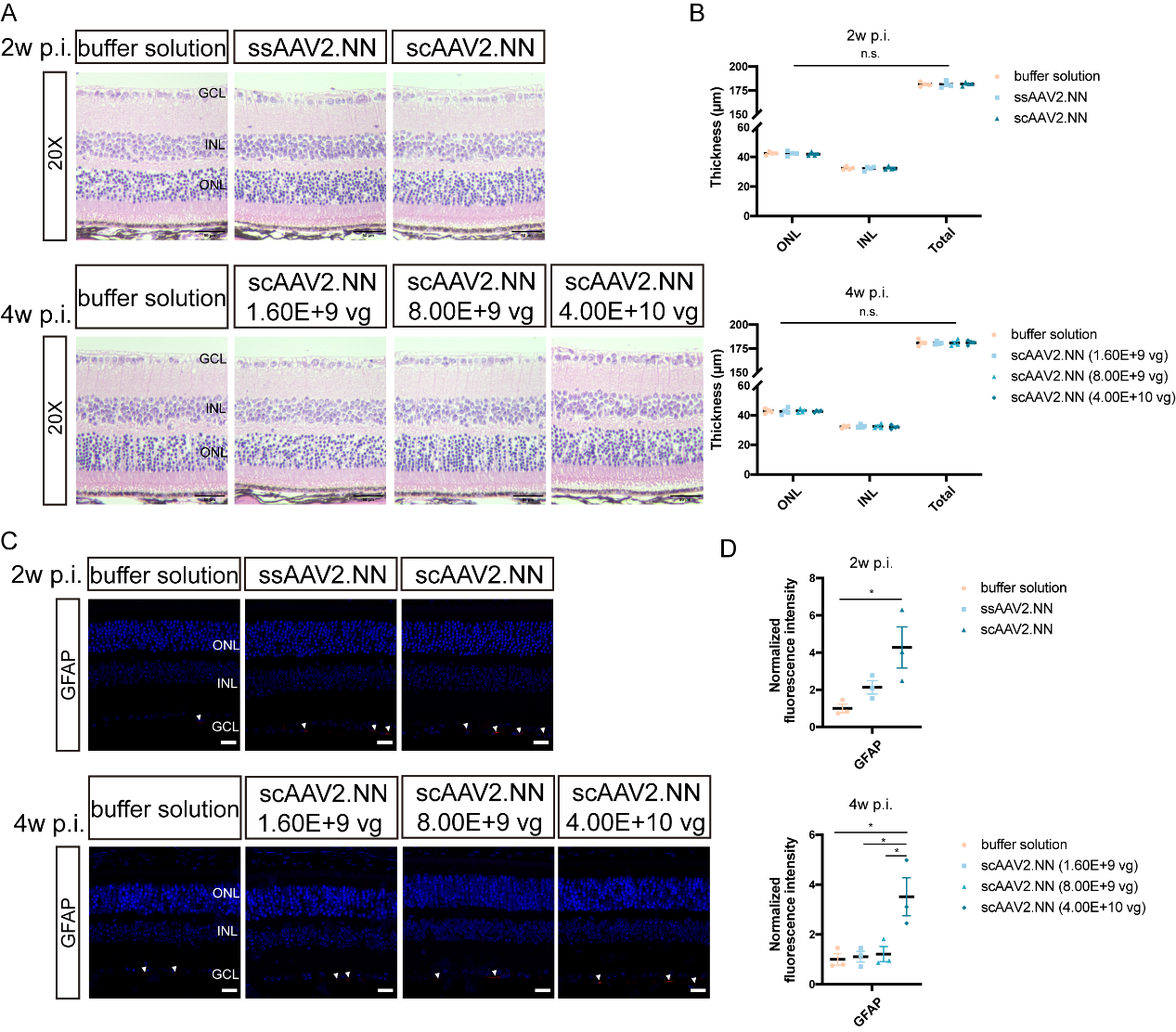


**Figure S3. The comparison of retinal structure and GFAP fluorescence in retinas transduced with ssAAV2.NN and scAAV2.NN vectors at a 5-fold dose gradient respectively 2 and 4 weeks after intravitreal injection.** (**A**) The H&E staining of retinas transduced with ssAAV2.NN, scAAV2.NN vectors at a dose gradient, and buffer solution respectively 2 and 4 weeks after intravitreal injection, with a scale bar of 50 μm. (**B**) The comparison of ONL, INL, and total thickness among retinas transduced with ssAAV2.NN, scAAV2.NN vectors at a dose gradient, and buffer solution respectively 2 and 4 weeks after intravitreal injection. (**C**) The immunofluorescence staining of GFAP of retinas transduced with ssAAV2.NN, scAAV2.NN vectors at a dose gradient, and buffer solution respectively 2 and 4 weeks after intravitreal injection. The red fluorescence refers to GFAP and the blue represents cell nuclei, with a scale bar of 20 μm. The white arrows indicate GFAP expression. (**D**) The comparison of normalised GFAP fluorescence intensity among retinas transduced with ssAAV2.NN, scAAV2.NN vectors at a dose gradient, and buffer solution respectively 2 and 4 weeks after intravitreal injection. * *P*<0.05, n.s., no significant differences, n≥3 in each group. GFAP: glial fibrillary acidic protein; AAV: adeno-associated virus; H&E: hematoxylin-eosin staining; ONL: outer nuclear layer; INL: inner nuclear layer

**
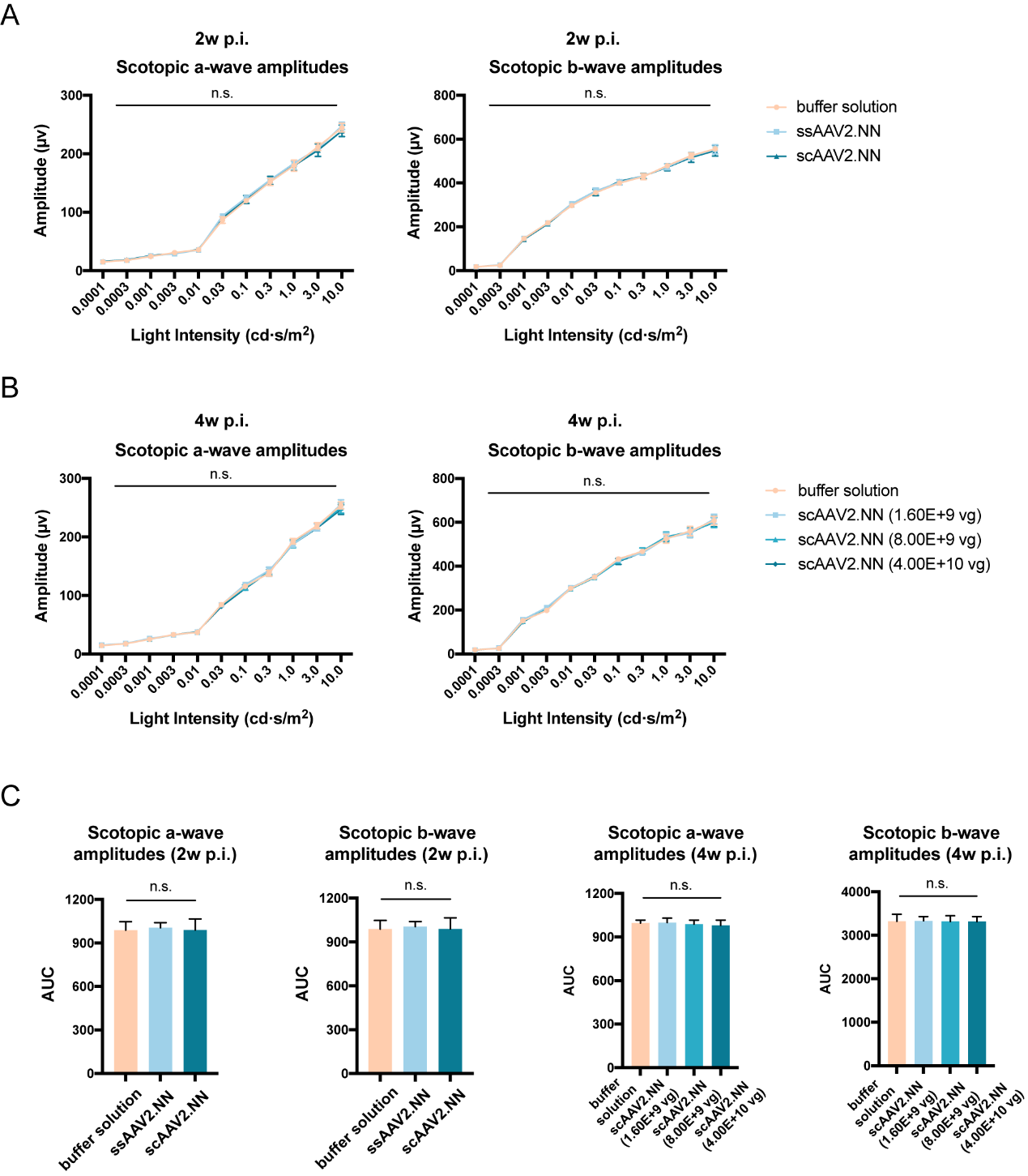
Figure S4. The comparison of visual function in mice intravitreally injected with ssAAV2.NN and scAAV2.NN vectors at a 5-fold dose gradient respectively 2 and 4 weeks after administration tested by ERG recordings.** (**A**) The comparison of a-wave and b-wave amplitudes among mice intravitreally injected with ssAAV2.NN, scAAV2.NN vectors, and buffer solution under the scotopic condition 2 weeks after administration. (**B**) The comparison of a-wave and b-wave amplitudes among mice intravitreally injected with scAAV2.NN vectors at a dose gradient and buffer solution under the scotopic condition 4 weeks after administration. (**C**) The comparison of AUC of a-wave and b-wave amplitudes among mice intravitreally injected with ssAAV2.NN, scAAV2.NN vectors at a dose gradient, and buffer solution under the scotopic condition respectively 2 and 4 weeks after administration. n.s., no significant differences, n=4 in each group.

**Abbreviations**

| Abbreviations | Full name |
| --- | --- |
| AAV | adeno-associated virus |
| BCs  BSA  CNS | bipolar cells  bovine albumin fraction V  central nervous system |
| GCL | ganglion cell layer |
| GFAP | glial fibrillary acidic protein |
| GS | glutamine synthetase |
| HCs  H&E | horizontal cells  hematoxylin-eosin |
| INL | inner nuclear layer |
| MCs  NIH | Müller cells  National Institutes of Health |
| ONL | outer nuclear layer |
| PBS | phosphate-buffered saline |
| PFA | paraformaldehyde |
| rAAV  RBCs  RGCs | recombinant adeno-associated virus  rod bipolar cells  retinal ganglion cells |
| RPE | retinal pigment epithelium |
